# Optimal Cutoff Size of Large Borrmann Type III Gastric Cancer: Is 8 cm Accurate in Predicting Survival and Incidence of Peritoneal Metastasis?

**DOI:** 10.1002/ags3.70071

**Published:** 2025-07-31

**Authors:** Yutaka Sugita, Manabu Ohashi, Naoki Miyazaki, Motonari Ri, Rie Makuuchi, Tomoyuki Irino, Masaru Hayami, Takeshi Sano, Souya Nunobe

**Affiliations:** ^1^ Department of Gastroenterological Surgery Cancer Institute Hospital, Japanese Foundation for Cancer Research Tokyo Japan; ^2^ Division of Clinical Research Planning and Strategy Cancer Institute Hospital, Japanese Foundation for Cancer Research Tokyo Japan

**Keywords:** Borrmann type III, gastric cancer, peritoneal metastasis, staging laparoscopy, survival outcome

## Abstract

**Background:**

Large type III gastric cancer (GC) ≥ 8 cm has conventionally been categorized with type IV GC in Japan, leading to alternative treatment strategies such as neoadjuvant chemotherapy and staging laparoscopy (SL). However, whether 8 cm is the correct cutoff remains unclear.

**Methods:**

We retrospectively analyzed patients clinically diagnosed with advanced GC who underwent surgery at our department. Patients were classified by Borrmann type, and clinicopathological characteristics including survival outcomes and peritoneal metastasis incidence were analyzed based on tumor size to determine the optimal cutoff for large type III GC.

**Results:**

Tumor size correlated with overall survival in type III GC. Although hazard ratios (HRs) for “large” and “small” type III vs. type IV remained comparable up to the 8 cm cutoff (0.60 and 0.41, respectively), HR for “large” type III GC increases sharply to 0.74 with a 10 cm cutoff. Subgroup analysis based on histological subtype revealed similar results in the undifferentiated type. Conversely, a larger cutoff value appeared more appropriate for the differentiated type. The largest difference in the incidence of peritoneal metastasis was observed with a 6 cm cutoff (36.1% in “large” type III and 10.2% in “small” type III), and similar results were observed in the undifferentiated type at the same cutoff.

**Conclusions:**

In terms of survival, a 10 cm cutoff may more accurately define large type III GC than the conventional 8 cm. However, if surgeons intend to identify peritoneal metastasis by SL, type III GC ≥ 6 cm could be a possible candidate.

## 1 Introduction

Despite the steady decline in gastric cancer (GC) incidence over the last 60 years, it was the fourth leading cause of cancer‐associated death worldwide in 2020 [1]. Advancements in chemotherapy and surgical techniques have led to improved survival outcomes of GC, but those of locally advanced GC remain unsatisfactory [2, 3]. Therefore, it is important to choose the most appropriate therapeutic strategy according to the characteristics of each tumor.

Locally advanced GC is classified into the following four groups according to macroscopic findings: Borrmann type I as mass type; type II as ulcerative type; type III as infiltrative ulcerative type; and type IV as diffuse infiltrative type [4]. Type IV GC, which is called linitis plastica, is known to have poorer survival and a higher incidence of peritoneal metastasis than the other types [5, 6]. Type III GC ≥ 8 cm in diameter was reported to have oncological characteristics similar to type IV GC and is referred to as “large type III GC” [7]. Because of these characteristics, large type III and type IV GCs have been categorized differently to other types of GC and other treatment strategies have been developed in Japan [8, 9]. Neoadjuvant chemotherapy (NAC) followed by gastrectomy has been investigated as a promising strategy for large type III and type IV GC, while a phase III trial is currently underway to evaluate the efficacy of NAC in other GC types. Furthermore, according to the Japanese Gastric Cancer Treatment Guidelines, staging laparoscopy (SL) is weakly recommended for patients with large type III or type IV GC because of the high incidence of peritoneal metastasis [10].

A tumor diameter cutoff size of 8 cm for large type III GC was first proposed in 1992 [7]. Since then, the survival outcomes of large type III and type IV GCs have improved with the introduction of SL and adjuvant chemotherapy, which have narrowed the gap with other Borrmann types. Despite such significant changes, the optimal cutoff size for large type III GC has not been thoroughly validated. Hosoda et al. reported that a preoperative tumor size exceeding 5.3 cm was an independent prognostic factor for overall survival (OS) in patients with type III GC with negative peritoneal cytology (CY0). However, such patients had a much better survival outcome than those with type IV GC [11], and whether it is the most accurate cutoff size to distinguish large type III and type IV from the other GC types remains unclear.

To clarify whether 8 cm is a suitable tumor diameter cutoff size and to determine the optimal cutoff size of large type III GC, we retrospectively analyzed survival outcomes and the incidence of peritoneal metastasis according to the Bormann classification using patients who were treated with current strategies for locally advanced GC. The results obtained from this study will contribute to the development of a new treatment strategy for locally advanced GC.

## 2 Patients and Methods

### 2.1 Patients

**FIGURE 1:**
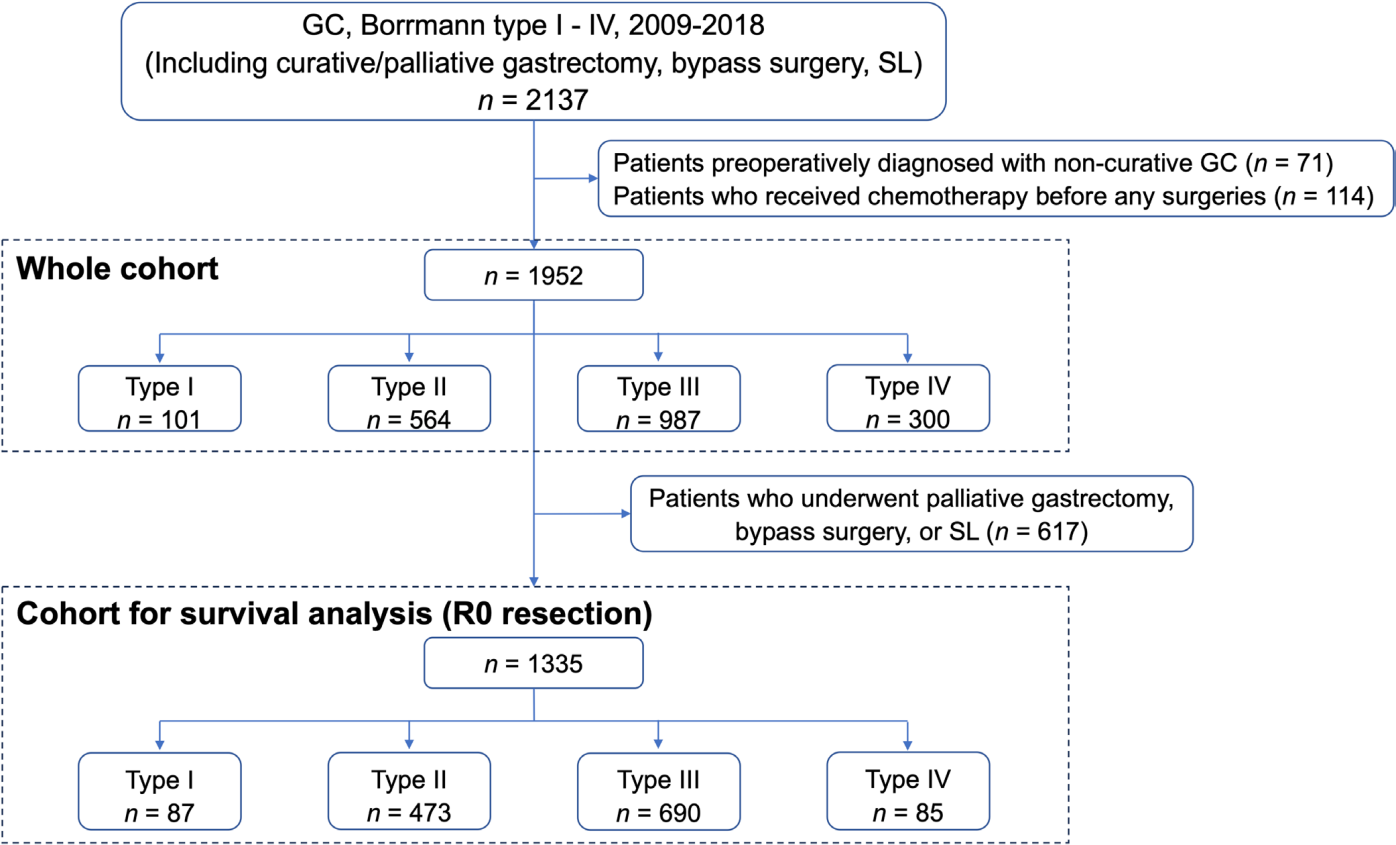
Flowchart of the study design. GC, gastric cancer; SL, staging laparoscopy.

Consecutive GC patients clinically diagnosed with Borrmann type I to IV, who underwent surgery including curative/palliative gastrectomy, bypass surgery, and SL at the Cancer Institute Hospital between 2009 and 2018, were enrolled in this study. Patients preoperatively diagnosed with non‐curative GC, or who received chemotherapy before any surgeries were excluded, and the remaining 1952 patients were retrospectively analyzed (whole cohort). Patients were classified into four groups according to Borrmann classification. For the survival outcome analysis, patients who underwent bypass surgery or SL and patients with distant metastasis or R1/R2 resections were excluded, and the remaining 1335 patients with R0 resection were analyzed for each Borrmann type (cohort for survival analysis) (Figure 1).

The present study was approved by the ethics committee of the Cancer Institute Hospital in accordance with the Declaration of Helsinki. The experimental protocols met the guidelines for clinical research at the Cancer Institute Hospital, and informed consent was obtained from all patients using the opt‐out recruitment method.

### 2.2 Assessment of Clinicopathological Characteristics

Clinicopathological findings, intraoperative data, and survival outcomes were retrospectively extracted from our prospectively developed database. All patients were staged preoperatively using gastroscopy (GS) and enhanced computed tomography. The clinical Borrmann type was determined based on the gross findings of the preoperative GS. The clinical tumor diameter was measured using an endoscopic ruler for smaller lesions. In patients with larger tumors, the diameter of the endoscope (approximately 1 cm) was used as a reference, and additional estimations were made by rotating the endoscope and referring to the calibration markings on the scope. Estimated tumor diameters were recorded in the official endoscopic examination reports. In patients whose tumor size had not been recorded, the surgeons determined the diameter based on a comprehensive assessment of the upper gastrointestinal series and GS findings. The staging was determined based on the 15th edition of the Japanese Classification of Gastric Carcinoma [4]. Well‐ or moderately‐differentiated tubular adenocarcinoma and papillary adenocarcinoma are defined as differentiated histological type, while undifferentiated type comprised solid and non‐solid types of poorly differentiated adenocarcinoma, signet‐ring cell carcinoma, and mucinous adenocarcinoma. At our institution, SL is indicated for patients with potentially resectable large type III tumors ≥ 8 cm in diameter, type IV tumors, bulky lymph node, or paraaortic lymph node involvement. OS was defined as the time from the date of surgery to death from any cause. Patients who were lost to follow‐up or were still alive at the time of evaluation were censored.

### 2.3 Treatment for Advanced Gastric Cancer

Following the weak recommendation provided by the Japanese Gastric Cancer Treatment Guidelines [10], NAC was administered to patients with extensive lymph node metastasis in the regional and paraaortic lymph nodes. Some patients received NAC as participants in certain clinical studies [9, 12]. Gastrectomy with D2 lymphadenectomy was routinely performed for patients with advanced GC. The resection margin was routinely checked by frozen pathological examination during the surgery. In patients with a positive resection margin, additional resections were performed with curative intent. For potential curative resection, combined organ resection was selectively performed. Patients with pathological stage II or III received adjuvant chemotherapy, taking into consideration the patient's willingness and physical condition. Most patients received S‐1 monotherapy as the most common regimen in Asia, and DS (S‐1 plus docetaxel) was introduced in 2018 for patients with pathological stage III. Other regimens were chosen based on the pathological findings or clinical trials [13, 14, 15].

### 2.4 Assessment of Peritoneal Metastasis

A diagnosis of peritoneal metastasis including gross peritoneal deposit (P1) and positive peritoneal cytology (CY1) was histologically assessed using an intraoperatively collected specimen. P1 was further subdivided into P1a, P1b, and P1c based on the 15th edition of the Japanese Classification of Gastric Carcinoma [4]. In the present study, the incidence of peritoneal metastasis was primarily defined as the proportion of patients with CY1 or P1.

### 2.5 Evaluation of the Optimal Cutoff Size for Large Type III GC in Survival

The purpose of categorizing the types of GC is to distinguish GC with poor survival from GC with more standard survival. When a cutoff is determined for large or small type III, the following conditions are ideal: the survival outcome of large type III GC is close to that of type IV GC and the survival outcome of small type III GC is close to that of type I/II GC. To determine the optimal cutoff size for large type III GC based on the survival outcome, we set cutoff values at intervals, ranging from 4 to 12 cm, and evaluated the hazard ratio (HR) of “large” and “small” type III GCs at each cutoff value as well as type I/II GCs, which were compared to type IV GC. To evaluate whether 8 cm was the optimal cutoff or not, we first calculated the HRs for “large” and “small” type III GC using 8 cm as the cutoff value. Next, we calculated and compared the HRs for “large” and “small” type III GC at other cutoff values relative to those at 8 cm. Additionally, a subgroup analysis based on histological subtypes was performed.

### 2.6 Evaluation of the Optimal Cutoff Size for Large Type III GC in Peritoneal Metastasis

From the perspective of peritoneal metastasis detection, the optimal cutoff value for large type III GC should be the one that achieves the highest efficacy in identifying peritoneal metastasis. Therefore, we analyzed the correlation between tumor size and the presence of peritoneal metastasis using receiver operating characteristic (ROC) curve analysis. Following the determination of the optimal cutoff value, type III GCs were stratified using that cutoff and 8 cm, and the incidence of peritoneal metastasis was compared between the group consisting of type IV and “large” type III GCs and the group comprising other types and “small” type III GCs. Furthermore, a subgroup analysis was conducted based on histological subtypes as well as subtypes of peritoneal metastasis.

### 2.7 Statistical Methods

Data were expressed as median and range for continuous variables, and as frequency and percentage for categorical variables. Differences between groups were analyzed using the chi‐squared test with odds ratios (ORs) and 95% confidence intervals (CIs). The association between a continuous variable and a binary outcome was assessed by ROC curve analysis. A time‐dependent ROC analysis was performed to evaluate the association between a continuous variable and survival outcome. Survival curves of OS were generated using the Kaplan–Meier method. The overall differences between survival curves were compared using the Cox proportional hazards model to estimate HRs and 95% CIs. All statistical analyses were conducted using JMP software (SAS Institute, Cary, NC, USA). Values of *p* < 0.05 were considered significant.

## 3 Results

### 3.1 Clinicopathological Characteristics of Each Clinical Borrmann Type

The characteristics of 1952 patients in whole cohort and 1335 patients with R0 resection are summarized in Table 1. The median follow‐up period was 53.7 months in the whole cohort. In the survival analysis (patients with R0 resection), the median follow‐up period was 61.4 months and the rate of patients lost to follow‐up was 3.1%. In the whole cohort, the incidence of peritoneal metastasis including CY1 or P1 was 4.0% for type I, 6.2% for type II, 21.8% for type III, and 64.0% for type IV. The histological type showed a predominance of differentiated tumors in type I and type II, while poorly differentiated tumors were more common in type III and type IV. In patients with R0 resection, pathological T and N grades tended to be more advanced in type III and type IV GCs, and a higher proportion of these patients received adjuvant chemotherapy.

**TABLE 1:** Clinicopathological characteristics of each clinical Borrmann type for whole cohort and cohort for survival analysis.

	Whole cohort (*n* = 1952)				Cohort for survival analysis (*n* = 1335)			
	Type I (*n* = 101)	Type II (*n* = 564)	Type III (*n* = 987)	Type IV (*n* = 300)	Type I (*n* = 87)	Type II (*n* = 473)	Type III (*n* = 690)	Type IV (*n* = 85)
Age^a^	70 (39–92)	69 (23–89)	65 (22–95)	63 (21–89)	70 (39–92)	69 (23–89)	66 (22–95)	61 (26–87)
Sex (%)								
Female	21 (20.8)	174 (30.9)	333 (33.7)	138 (46.0)	19 (21.8)	147 (31.1)	218 (31.6)	38 (44.7)
Male	80 (79.2)	390 (69.1)	654 (66.3)	162 (54.0)	68 (78.2)	326 (68.9)	472 (68.4)	47 (55.3)
Liver metastasis (%)								
H0	100 (99.0)	542 (96.1)	961 (98.4)	278 (99.3)	87 (100)	473 (100)	690 (100)	85 (100)
H1	1 (1.0)	22 (3.9)	16 (1.6)	2 (0.7)	0 (0)	0 (0)	0 (0)	0 (0)
CY1 or P1 (%)								
Negative	97 (96.0)	529 (93.8)	772 (78.2)	108 (36.0)	87 (100)	473 (100)	690 (100)	85 (100)
Positive	4 (4.0)	35 (6.2)	215 (21.8)	192 (64.0)	0 (0)	0 (0)	0 (0)	0 (0)
P0 CY1	2 (2.0)	15 (2.7)	76 (7.7)	73 (24.3)	0 (0)	0 (0)	0 (0)	0 (0)
P1a CYany	0 (0)	9 (1.6)	27 (2.7)	13 (4.3)	0 (0)	0 (0)	0 (0)	0 (0)
P1b/c CYany	2 (2.0)	11 (2.0)	112 (11.3)	106 (35.3)	0 (0)	0 (0)	0 (0)	0 (0)
Histological type (%)								
Undifferentiated	39 (38.6)	267 (47.3)	663 (67.2)	267 (90.2)	30 (34.5)	231 (48.8)	444 (64.3)	73 (85.9)
Differentiated	62 (61.4)	297 (52.7)	323 (32.8)	29 (9.8)	57 (65.5)	242 (51.2)	246 (35.7)	12 (14.1)
pT stage (%)^b^								
T0					0 (0)	2 (0.4)	0 (0)	0 (0)
T1a/b					28 (32.2)	52 (11.0)	55 (8.0)	4 (4.7)
T2					20 (23.0)	122 (25.8)	111 (16.1)	7 (8.2)
T3					19 (21.8)	142 (30.0)	175 (25.4)	9 (10.6)
T4a/b					20 (23.0)	155 (32.8)	349 (50.5)	65 (76.5)
pN stage (%)^b^								
N0					40 (46.0)	202 (42.7)	223 (32.3)	29 (34.1)
N1					26 (29.9)	123 (26.0)	137 (19.9)	8 (9.4)
N2					10 (11.5)	77 (16.3)	122 (17.7)	12 (14.1)
N3a/b					11 (12.6)	71 (15.0)	208 (30.1)	36 (42.4)
Neoadjuvant chemotherapy (%)								
Received					0 (0)	9 (1.9)	21 (3.0)	6 (7.1)
Not received					87 (100)	464 (98.1)	669 (97.0)	79 (92.9)
Adjuvant chemotherapy (%)								
Received					32 (37.2)	219 (46.4)	419 (60.7)	63 (74.1)
Not received					54 (62.8)	253 (53.6)	271 (39.3)	22 (25.9)

Abbreviations: CY, peritoneal lavage cytology; P, gross peritoneal deposit.

^a^ Data are expressed as median (range).

^b^ According to the Japanese Classification of Gastric Carcinoma.

### 3.2 Association Between Tumor Size and OS in Each Tumor Type

A time‐dependent ROC analysis revealed a consistent association between tumor size and OS in type III GC, while little association was observed for type I and type II (Figure S1).

### 3.3 Survival Curves of Type III GCs at Each Cutoff Value and the Other Types

**FIGURE 2:**
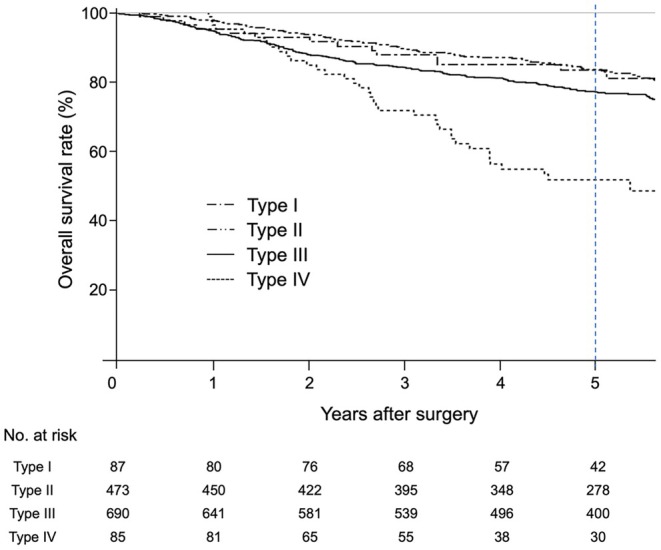
The survival curves of each Borrmann type reveal that the 5‐year OS rates are 83.5% (95% CI 73.1–90.1) for type I, 83.6% (95% CI 79.7–86.8) for type II, 77.1% (95% CI 73.7–80.2) for type III, and 51.8% (95% CI 39.8–62.6) for type IV GC. OS, overall survival; 95% CI, 95% confidence interval; GC, gastric cancer.

Figures 2 and S2 show the OS of each clinical Borrmann type in patients with R0 resection, as well as that for “large” and “small” type III GCs at each cutoff value with the other types. As shown in Table S1, among patients with type III GC, there was only one patient each with endoscopically measured tumor sizes of 9.0–9.9 cm and 11.0–11.9 cm, in contrast to measurements on resected specimens. Therefore, cutoffs greater than 8 cm were set at 2 cm intervals.

### 3.4 Optimal Cutoff Size for Large Type III GC in Survival Outcomes

#### 3.4.1 Primary Outcome

**FIGURE 3:**
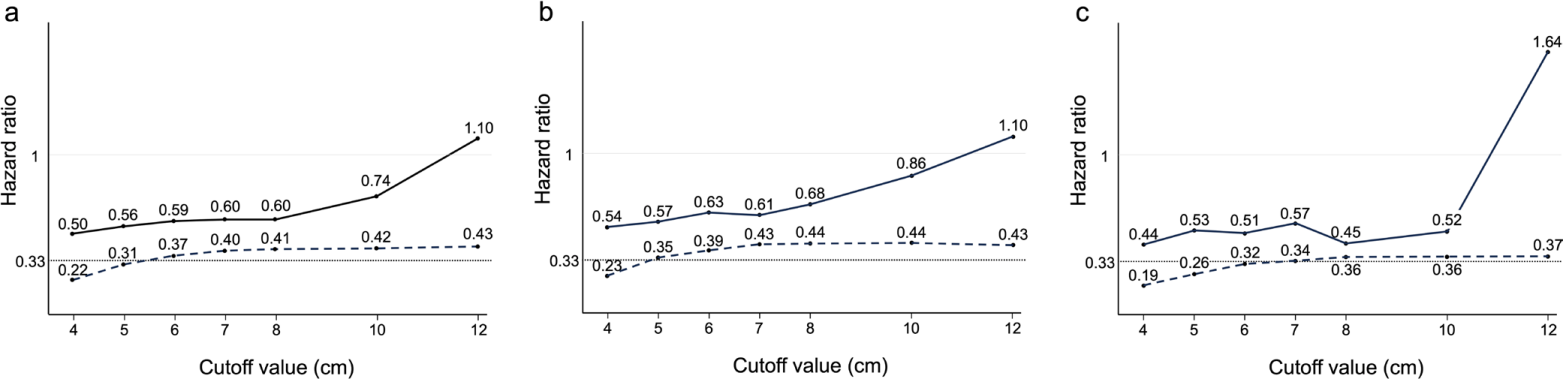
(a) HRs of “large” and “small” type III GCs at each cutoff value as well as type I/II GCs, which are compared to type IV GC. The HR of type I/II GCs is 0.33 (represented by a fine dotted line), and both the HR of “large” and “small” type III GCs increase as the cutoff value rises (solid line for “large” type III and dotted line for “small” type III). Subgroup analysis based on histological type are also shown for (b) undifferentiated type and (c) differentiated type. HR, hazard ratio; GC, gastric cancer.

Figure 3a shows the HRs of “large” and “small” type III GCs at each cutoff value as well as type I/II GCs, which were compared to type IV GC. The proportion of the corresponding patients and the 95% CI for the HR are also shown in Table S2. For the same reason as in the case of the survival curve, HRs were compared at 1 cm intervals up to 8 cm, and at 2 cm intervals for tumor sizes exceeding 8 cm. The HRs of both “large” and “small” type III GCs gradually increase as the cutoff value rises. When the cutoff value was set to 8 cm, the HR for “large” type III GC was 0.60 (95% CI 0.36–0.99) and that for “small” type GC was 0.41 (95% CI 0.29–0.59). They were almost the same when the cutoff value was set to 7 cm, and the HR for “large” type III GC decreased with the cutoff value less than 7 cm. However, when the cutoff value was set to 10 and 12 cm, the HRs for “large” type III GC were 0.74 (95% CI 0.41–1.33) and 1.10 (95% CI 0.39–3.09), respectively, while those for “small” type III GC were still 0.42 to 0.43.

#### 3.4.2 Subgroup Analysis Based on Histological Type

In undifferentiated‐type tumors, the HR for “large” type III GC gradually increased from 8 cm, reaching 0.86 at 10 cm (Figure 3b). In contrast, in differentiated‐type tumors, the HR remained relatively constant at approximately 0.5 up to a cutoff value of 10 cm (Figure 3c).

### 3.5 Optimal Cutoff Size for Large Type III GC in Peritoneal Metastasis

#### 3.5.1 Primary Outcome

**FIGURE 4:**
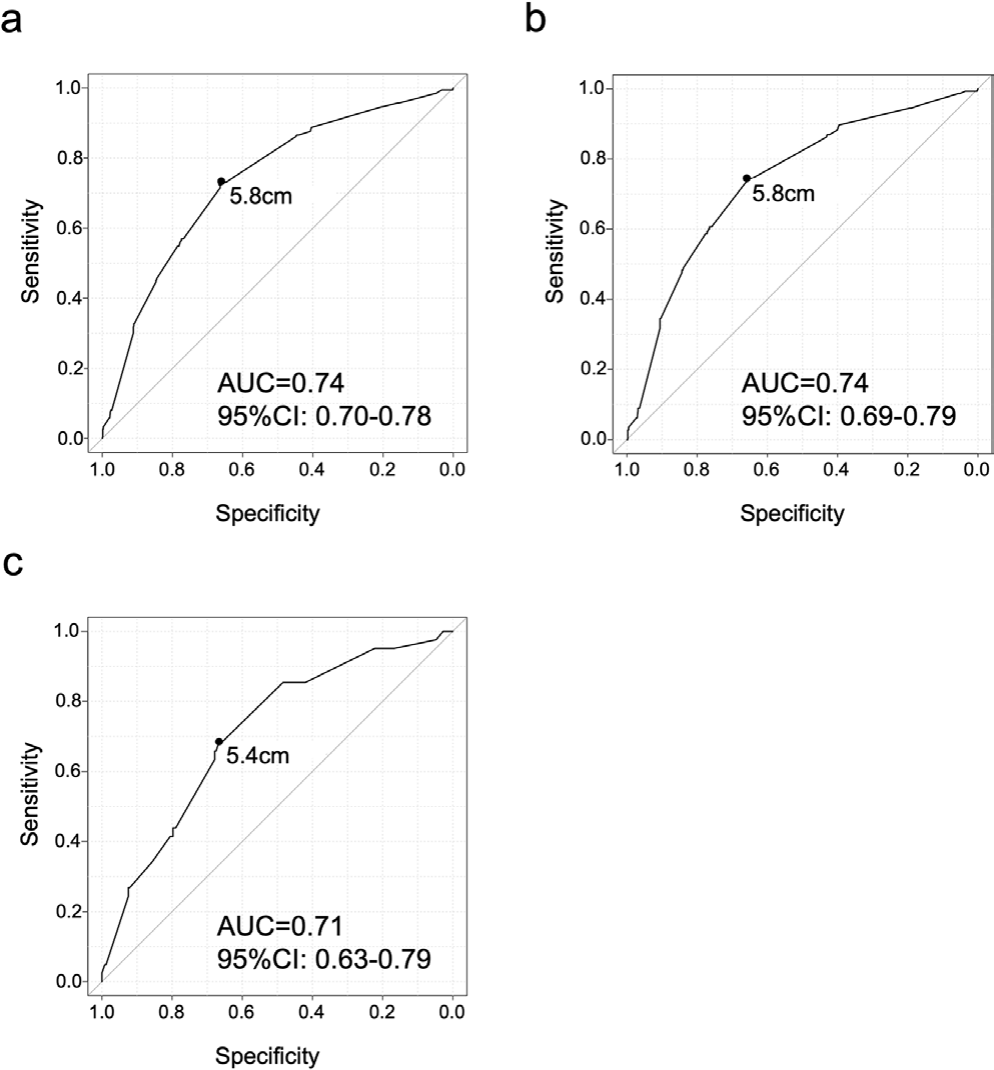
A ROC curve analysis between the incidence of peritoneal metastasis and the tumor size of all type III GC (a), undifferentiated type III GC (b), and differentiated type III GC (c). ROC, receiver operating characteristic; GC, gastric cancer; AUC, area under the curve; 95% CI, 95% confidence interval.

**FIGURE 5:**
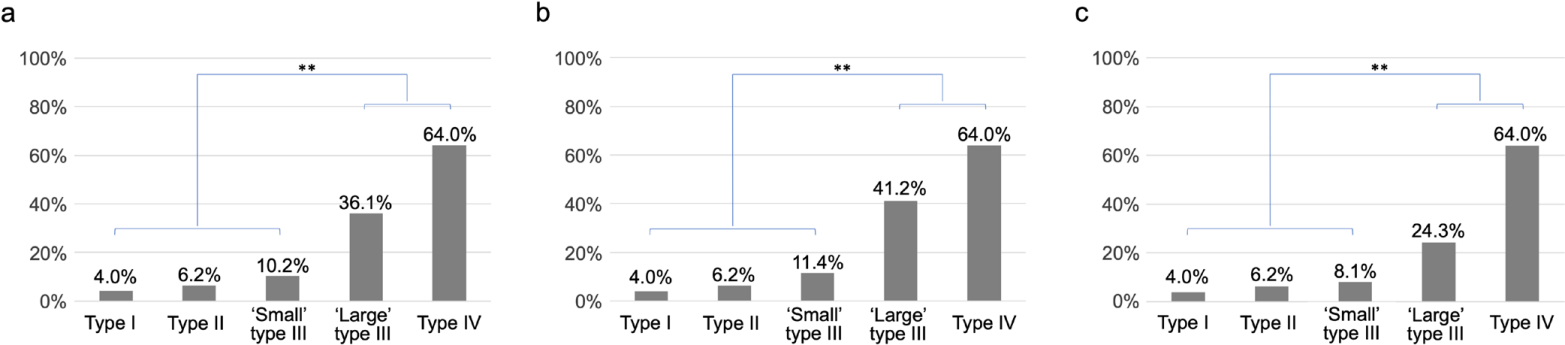
The incidences of peritoneal metastasis in “large” and “small” type III GCs with other Borrmann types, at a cutoff of 6 cm for all type III GC (a), undifferentiated type (b), and differentiated type (c). GC, gastric cancer; **p < 0.01.

The incidence of peritoneal metastasis according to tumor size in each Borrmann type is shown in Table 2. For type III GC, detailed results are additionally provided according to histological type. The incidence of peritoneal metastasis in type III GC was higher than that for the same tumor size in type I/II GCs, especially in the undifferentiated type. An AUC value of 0.74 (95% CI 0.70–0.78) was observed for the ROC curve between the tumor size of type III GC and the incidence of peritoneal metastasis in the whole cohort, and the corresponding cutoff size was 5.8 cm (Figure 4a). Following this result, a cutoff of 6 cm was set for large type III GC, and the incidences of peritoneal metastasis were 36.1% in “large” type III GC and 10.2% in “small” type III GC (Figure 5a). The chi‐squared test revealed that the incidences of peritoneal metastasis for type I, type II, and “small” type III were significantly lower than those for “large” type III and type IV (7.8% vs. 48.7%, OR = 11.2, 95% CI 8.6–14.7). This trend was consistent even with the conventional tumor diameter cutoff of 8 cm (10.4% vs. 56.1%, OR = 11.0, 95% CI 8.5–14.2) (Figure S3).

**TABLE 2:** Incidence of peritoneal metastasis according to tumor size for each Borrmann type and also histological type for type III GC.

Tumor size (cm)	Type I		Type II		Type III					
					All		Undifferentiated		Differentiated	
4.0 ≤	3/57	(5.3%)	31/349	(8.9%)	176/733	(24.0%)	137/497	(27.6%)	39/235	(16.6%)
5.0 ≤	2/34	(5.9%)	22/221	(10.0%)	160/545	(29.4%)	125/379	(33.0%)	35/165	(21.2%)
6.0 ≤	0/16	(0%)	16/130	(12.3%)	133/368	(36.1%)	107/260	(41.2%)	26/107	(24.3%)
7.0 ≤			13/79	(16.5%)	102/252	(40.5%)	85/185	(45.9%)	17/66	(25.8%)
8.0 ≤			8/47	(17.0%)	83/190	(43.7%)	69/139	(49.6%)	14/50	(28.0%)
9.0 ≤			4/28	(14.3%)	60/123	(48.8%)	49/91	(53.8%)	11/31	(35.5%)
10.0 ≤					56/118	(47.5%)	46/88	(52.3%)	10/29	(34.5%)
11.0 ≤					14/30	(46.7%)	12/26	(46.2%)	2/4	(50.0%)
12.0 ≤					11/25	(44.0%)	9/21	(42.9%)	2/4	(50.0%)

#### 3.5.2 Subgroup Analysis Based on Histological Type

In undifferentiated‐type III GC, ROC curve analysis between the tumor size and the incidence of peritoneal metastasis revealed the similar results as all type III GC, with an AUC value of 0.74 (95% CI 0.69–0.79) and the corresponding cutoff size of 5.8 cm (Figure 4b), and the incidence of 41.2% in “large” group and 11.4% in “small” group using 6 cm cutoff (Figure 5b). In differentiated‐type III GC, ROC curve analysis revealed the most accurate cutoff value as 5.4 cm, but the incidence of peritoneal metastasis remained at 24.3% even in the “large” group using 6 cm cutoff (Figures 4c and 5c).

#### 3.5.3 Subgroup Analysis for Detailed Peritoneal Metastasis

The incidence of peritoneal metastasis was additionally evaluated in the cohort excluding P0CY1 patients (peritoneal metastasis was defined as P1CYany), as well as in a further refined subgroup excluding P1aCYany patients (peritoneal metastasis was defined as P1b/cCYany) from the original cohort of patients with peritoneal metastasis. Using ROC curve analysis, the optimal cutoff size for detecting peritoneal metastasis was determined for both groups and was found to be 5.8 cm, identical to the value obtained in the primary outcome (Figure S4a,b). The incidence of peritoneal metastasis in each type of GC remained essentially consistent with that of the original analysis across both cohorts (Figure S4c,d).

## 4 Discussion

In this retrospective study, we investigated whether a tumor diameter of 8 cm is an appropriate cutoff size for large or small type III GC to distinguish between GC with poor and standard survival, and, if not, to decide the optimal cutoff size for survival and peritoneal metastasis incidence. We identified three new findings. First, the survival outcome of type III GC depends on the tumor size. It may be proper that type III GC is classified according to the size of tumor. Second, the survival outcome of “large” type III GC clearly worsened when a cutoff value ≥ 10 cm was applied. This finding suggests that the optimal cutoff value should be larger than the conventional 8 cm, with 10 cm being more appropriate. Third, a cutoff size of 6 cm resulted in the largest difference in peritoneal metastasis incidence. If surgeons intend to identify peritoneal metastasis by SL, type III GC ≥ 6 cm could be a possible candidate.

When we determine the treatment strategy according to tumor size, only type III GC may be suitable. Several previous studies reported that both the depth of wall invasion and lymph node metastasis increased with a larger tumor size and correlated with survival outcome; however, most of these studies focused on pathological tumor size [16, 17, 18, 19] and no studies investigated the gross type. When considering preoperative therapy for GC with poor survival outcomes, it is not possible to determine the pathological tumor size before surgery, especially that of infiltrative types such as type III and IV. Thus, we must focus on the clinical tumor size to obtain useful information in daily clinical practice. In the present study, it was particularly intriguing that the survival outcomes of patients with R0 resection correlated with tumor size only for type III GC but not for the other macroscopic types. As we demonstrated, the incidence of peritoneal metastasis in type III GC was higher than that in type I/II GCs, despite them being of the same tumor size, suggesting a higher incidence of peritoneal recurrence after curative resection. Additionally, in type III GC, as the tumor size increased, the incidence of peritoneal metastasis also increased; this indicates a potential correlation between tumor size and the incidence of peritoneal recurrence and would result in a correlation between tumor size and survival outcome in type III GC.

In Japan, large type III GC ≥ 8 cm in size has long been considered the optimal cutoff value. Such disease has been thought to have a poor survival outcome equivalent to type IV GC; hence, these two GCs are categorized together and have been subject to a distinct treatment strategy. This cutoff value of 8 cm was proposed based on a report published by Sasako et al. in 1992 [7]. In their study, they examined the 5‐year OS rates after curative resection in patients with GCs of the following sizes for each macroscopic type: less than 4 cm; 4 cm or more but less than 8 cm; 8 cm or more but less than 12 cm; and 12 cm or more. For type III GC, the 5‐year OS rate was 36.8% for 8 cm or more but less than 12 cm, and 0% for 12 cm or more. For type IV GC, the rate was 26.1% for 8 cm or more but less than 12 cm, and 12.9% for 12 cm or more. In the present study, the 5‐year OS rate was 72.3% for type III GC ≥ 8 cm and 51.8% for type IV GC. Survival outcomes have significantly improved over the past 30 years and are attributed not only to advancements in surgical techniques and devices but also to the implementation of SL and adjuvant chemotherapy. However, these factors contributing to improved survival outcomes ironically highlight the difference between large type III and type IV GCs. Considering the substantial difference in survival outcomes between type III GC ≥ 8 cm and type IV GC, it is considered necessary to establish a new cutoff value for large type III GC given the current circumstances.

Type III GC is expected to increasingly resemble the characteristics of type IV GC as it grows larger due to its invasive nature. In the present study, the HR of “large” type III GC relative to type IV did not differ between the cutoff values of 7 and 8 cm. However, when the cutoff was increased in 2‐cm increments beyond 8 cm, the HR gradually rose, while the HR of “small” type III GC remained stable. This suggests that a cutoff value larger than the conventional 8 cm may be more appropriate. Nevertheless, since the HR increases gradually with tumor size, determining the optimal new cutoff value remains a matter for discussion. In this study, there were very few patients with tumors in the 9 and 11 cm ranges, whereas those in the 10 cm and 12 cm ranges were more common. This indicates that assessing tumor size in 1‐cm increments for large GCs may be difficult and prone to inaccuracy. Therefore, we consider 10 cm to be a reasonable and practical cutoff value. While this represents only a 2 cm increase from the traditional standard, approximately 5.4% of patients would now be reclassified as having smaller tumors. We believe this reclassification has meaningful clinical implications, as it may lead to more appropriate, tumor‐specific treatment strategies. In the subgroup analysis based on the histological type, the HR began to increase from a smaller cutoff point in undifferentiated‐type tumors, whereas in differentiated‐type tumors, it remained lower and relatively constant until exceeding 10 cm. This finding is consistent with the most recent evidence [20], which reported that large undifferentiated type III GC (≥ 8 cm) shares similar oncological features with type IV GC. In the present study, if histological type is incorporated into the definition of large type III GC, cutoff of 10 cm and 12 cm would be applied to the undifferentiated and differentiated types, respectively. However, given the comparable results in both survival and peritoneal metastasis between all type III GC and the undifferentiated type, we concluded that there is no compelling rationale to restrict the definition of large type III GC to the undifferentiated type in the present study.

As mentioned above, the cutoff value for tumor size in type III GC has another significance in detecting peritoneal metastasis effectively because type III GC has peritoneal metastasis due to the tumor size being larger. GC with peritoneal metastasis is unresectable with curative intent, and systemic chemotherapy is indicated. In the Japanese Gastric Cancer Treatment Guidelines, SL is weakly recommended for large type III and type IV GCs because of the high incidence of peritoneal metastasis [10]. We investigated the optimal tumor size with regards to peritoneal metastasis incidence. In the ROC curve analysis in the present study, the tumor size that could most accurately identify peritoneal metastasis in type III GC was 5.8 cm. In fact, in type III GC ≥ 6 cm in size, the incidence of peritoneal metastasis was 36.1%, which ensured the cost‐effectiveness of SL [21], and these patients accounted for 41.5% of all type III GC patients. While the conventional indication for SL is type III GC ≥ 8 cm in size, in which the incidence of peritoneal metastasis was 43.7% and corresponding patients accounted for 21.4%, adopting this criterion may overlook a significant number of patients with peritoneal metastasis of type III GC with a tumor size between 6 and 8 cm. For these reasons, type III GC ≥ 6 cm in size may be a suitable candidate for SL, as well as type IV GC. Additionally, type I/II GCs, even with larger tumor sizes, have a lower incidence of peritoneal metastasis compared with type III, making them less likely to be candidates for SL.

The present study, while examining a relatively large number of patients, has limitations as a retrospective study conducted at a single institution, introducing the possibility of selection bias. Additionally, because of the infiltrative nature of type III GC with unclear boundaries, it was sometimes difficult to reproducibly measure tumor size and there is a potential for underestimating tumor size compared with type I/II GCs. In the current study, only 43 patients had type III GC ≥ 10 cm in size, accounting for 6.9% of all type III GCs in R0 resection cohort. Owing to this limited number of patients, the 95% CI for the HR was notably wide. Furthermore, only one patient each had tumors measuring 9 and 11 cm. This is likely due to the difficulty in accurately measuring large type III tumors endoscopically. In addition, in Japan, the distinction between tumors measuring ≥ 8 cm is considered clinically important, leading to a tendency for tumor sizes ≥ 8 cm to be recorded as rounded values. Based on these factors, tumor size assessments for tumors > 8 cm were performed in 2 cm increments. Further investigation with a larger number of patients may help identify a more appropriate cutoff value in the future. Finally, considering the macroscopic type of GC when selecting treatment strategies is a characteristic approach seen in Asia, including Japan, and thus the present findings may not be widely recognized on a global scale. However, in recent years, reports on the distinct features of type IV GC have emerged not only from Asia but also from Western countries [22, 23], and the notion that type IV GC represents a distinct subset from the more common types is increasingly being acknowledged internationally.

In conclusion, based on survival outcomes, an 8 cm cutoff does not appear to be appropriate for applying the same treatment strategy to large type III GC as to type IV GC; instead, a 10 cm cutoff may be more suitable, taking into account the ambiguity associated with clinical measurement of tumor size. On the other hand, from the viewpoint of peritoneal metastasis, type III GC of 6 cm or over in size should be considered as a candidate for SL, as well as type IV GC. The cutoff value for large type III GC in determining treatment strategies and that for SL indications were considered not necessarily required to be aligned. It is hoped that further large‐scale multicenter studies will validate the insights obtained in this study and contribute to the future management of GC.

## Author Contributions

**Yutaka Sugita:** conceptualization, investigation, writing – original draft, writing – review and editing, data curation, methodology. **Manabu Ohashi:** writing – review and editing, supervision, project administration, conceptualization, visualization. **Naoki Miyazaki:** methodology, formal analysis, data curation. **Motonari Ri:** supervision, validation. **Rie Makuuchi:** supervision, validation. **Tomoyuki Irino:** supervision, validation. **Masaru Hayami:** supervision, validation. **Takeshi Sano:** supervision, validation. **Souya Nunobe:** supervision, validation.

## Disclosure

This research did not receive any specific grant from funding agencies in the public, commercial, or not‐for‐profit sectors.

## Ethics Statement

The protocol for this research project has been approved by a suitably constituted Ethics Committee of the institution and it conforms to the provisions of the Declaration of Helsinki. Ethics Committee of Cancer Institute Hospital, Approval No. 2023‐GB‐092. All informed consent was obtained from the subjects.

## Conflicts of Interest

The authors declare no conflicts of interest.

## Supporting information


**FIGURE S1:** A time‐dependent ROC analysis between tumor size and OS for (a) type I, (b) type II, and (c) type III GCs. A consistent association is observed in type III GC (AUC 0.62 [95% CI 0.56–0.68] for 3‐year/AUC 0.63 [95% CI 0.58–0.68] for 5‐year), while little association is observed for type I (AUC 0.52 [95% CI 0.35–0.68] for 3‐year/AUC 0.59 [95% CI 0.42–0.72] for 5‐year) and type II (AUC 0.45 [95% CI 0.37–0.53] for 3‐year/AUC 0.53 [95% CI 0.45–0.59] for 5‐year).

**FIGURE S2:** The survival curves of “large” and “small” type III GCs at each cutoff value with other Borrmann types.

**FIGURE S3:** The incidences of peritoneal metastasis in “large” and “small” type III GCs with other Borrmann types at a conventional cutoff of 8 cm.

**FIGURE S4:** A ROC curve analysis between the tumor size of type III GC and the incidence of (a) P1CYany and (b) P1b/cCYany. The incidences of (c) P1CYany and (d) P1b/cCYany with other Borrmann types at a cutoff of 6 cm are also shown.

**TABLE S1:** Distribution of preoperative endoscopic and postoperative macroscopic tumor sizes in type III GC.
**TABLE S2:** HRs of “large” and “small” type III GCs compared to type IV GC at each cutoff value and the proportion of the corresponding patients.
