# Supplementary figures and images for: Optimal Cutoff Size of Large Borrmann Type III Gastric Cancer: Is 8 cm Accurate in Predicting Survival and Incidence of Peritoneal Metastasis?
Source: Ann Gastroenterol Surg. 2025 Jul 31;10(1):77–86. doi: 10.1002/ags3.70071 (PMC12757155; doi:10.1002/ags3.70071)

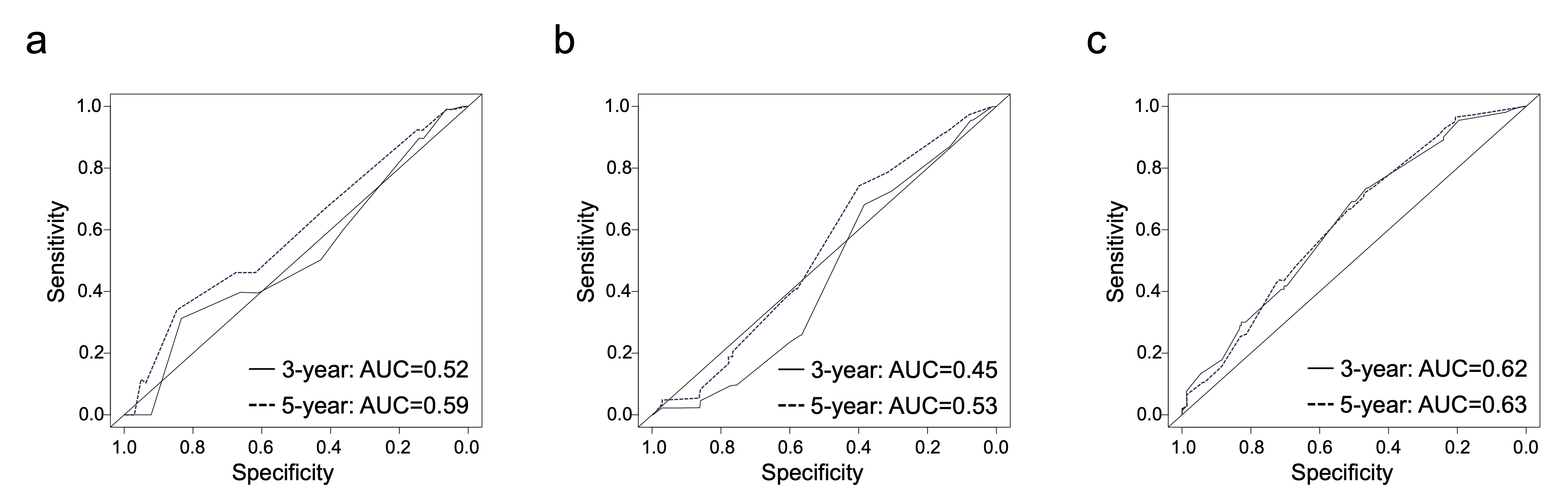

Supplement: Supplementary file 1 — FIGURE S1: A time‐dependent ROC analysis between tumor size and OS for (a) type I, (b) type II, and (c) type III GCs. A consistent association is observed in type III GC (AUC 0.62 [95% CI 0.56–0.68] for 3‐year/AUC 0.63 [95% CI 0.58–0.68] for 5‐year), while little association is observed for type I (AUC 0.52 [95% CI 0.35–0.68] for 3‐year/AUC 0.59 [95% CI 0.42–0.72] for 5‐year) and type II (AUC 0.45 [95% CI 0.37–0.53] for 3‐year/AUC 0.53 [95% CI 0.45–0.59] for 5‐year). [file AGS3-10-77-s004.tiff]

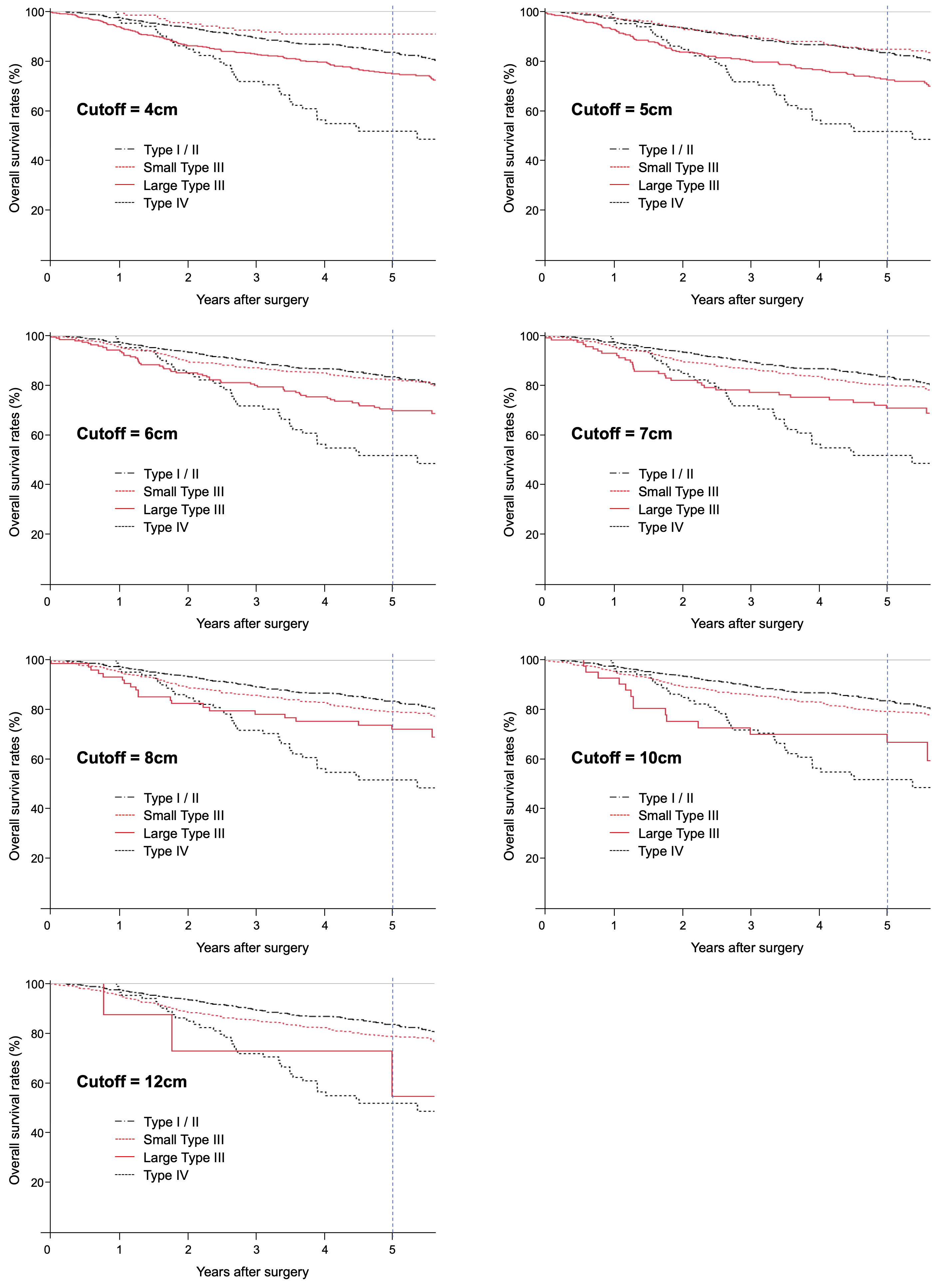

Supplement: Supplementary file 2 — FIGURE S2: The survival curves of “large” and “small” type III GCs at each cutoff value with other Borrmann types. [file AGS3-10-77-s001.tiff]

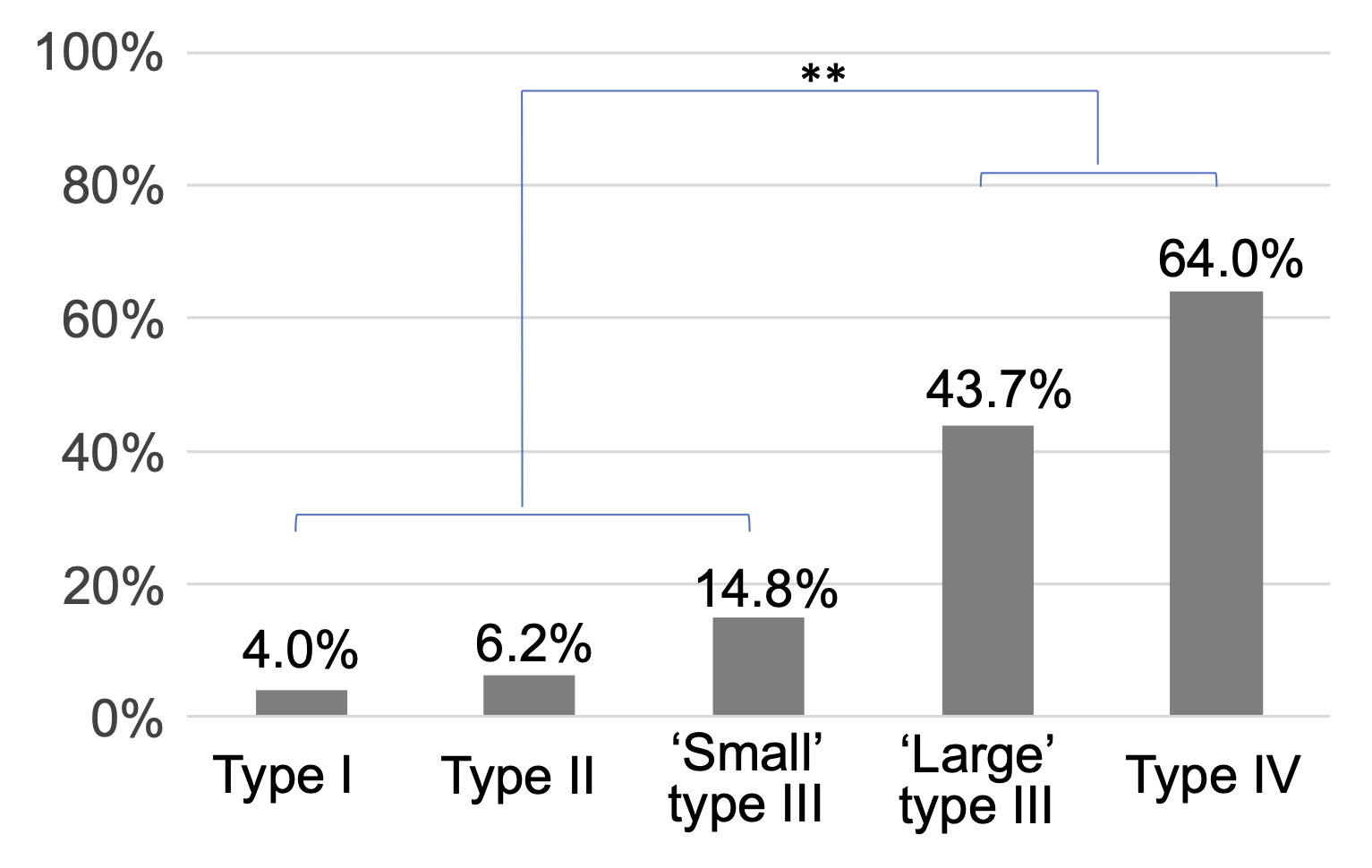

Supplement: Supplementary file 3 — FIGURE S3: The incidences of peritoneal metastasis in “large” and “small” type III GCs with other Borrmann types at a conventional cutoff of 8 cm. [file AGS3-10-77-s002.tiff]

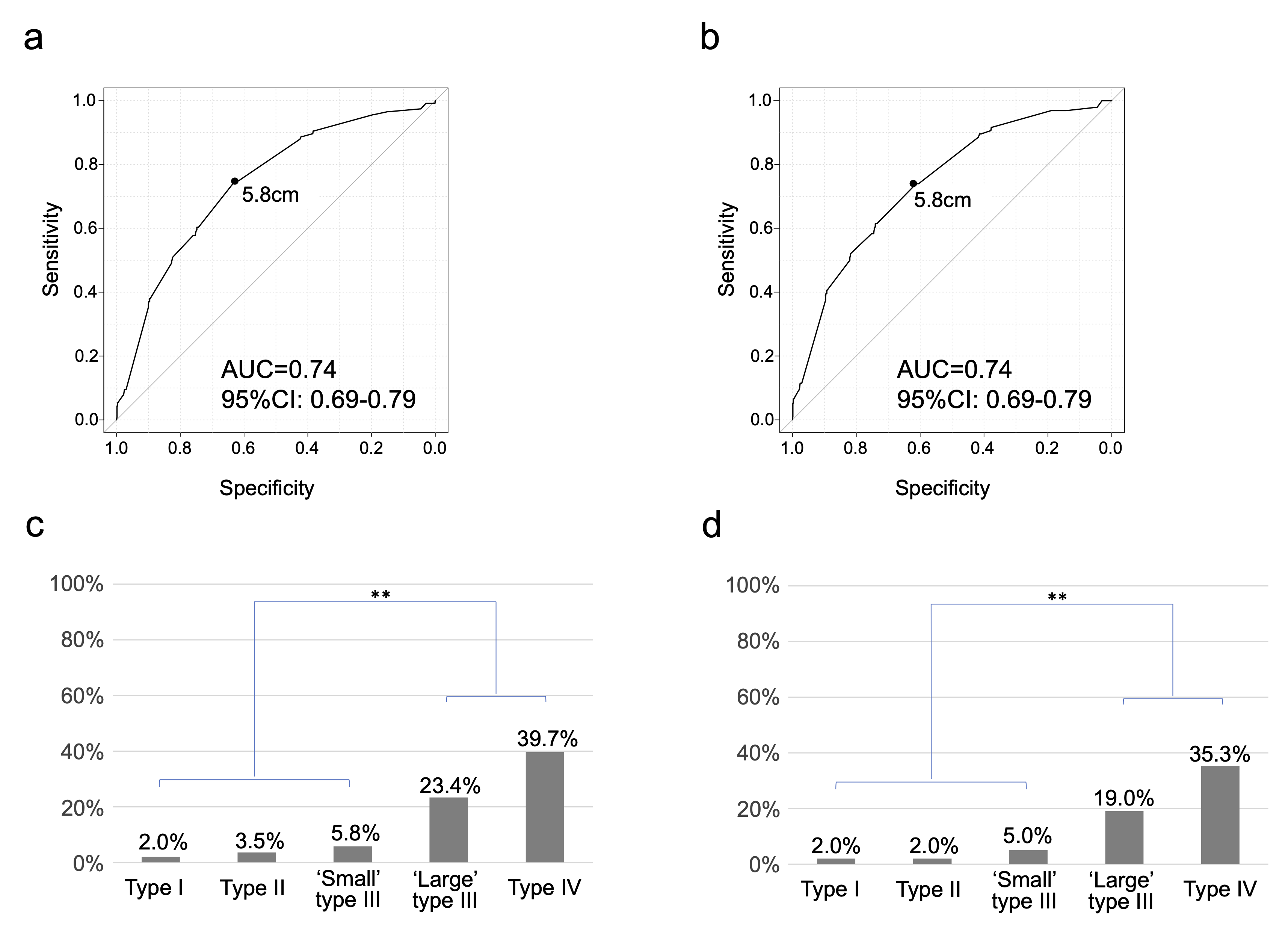

Supplement: Supplementary file 4 — FIGURE S4: A ROC curve analysis between the tumor size of type III GC and the incidence of (a) P1CYany and (b) P1b/cCYany. The incidences of (c) P1CYany and (d) P1b/cCYany with other Borrmann types at a cutoff of 6 cm are also shown. [file AGS3-10-77-s005.tiff]
