# Supplementary material for: Optimal Cutoff Size of Large Borrmann Type III Gastric Cancer: Is 8 cm Accurate in Predicting Survival and Incidence of Peritoneal Metastasis?
Source: Ann Gastroenterol Surg. 2025 Jul 31;10(1):77–86. doi: 10.1002/ags3.70071 (PMC12757155; doi:10.1002/ags3.70071)
Supplement: Supplementary file 5 — TABLE S1: Distribution of preoperative endoscopic and postoperative macroscopic tumor sizes in type III GC. TABLE S2: HRs of “large” and “small” type III GCs compared to type IV GC at each cutoff value and the proportion of the corresponding patients. [file AGS3-10-77-s003.docx]

Table S1. Distribution of preoperative endoscopic and postoperative macroscopic tumor sizes in type III GC

| Tumor size (cm) | Number of patients | |
| --- | --- | --- |
|  | Endoscopic measurement | Macroscopic measurement |
| 1.0 ≤, < 2.0 | 4 | 6 |
| 2.0 ≤, < 3.0 | 32 | 47 |
| 3.0 ≤, < 4.0 | 102 | 95 |
| 4.0 ≤, < 5.0 | 159 | 111 |
| 5.0 ≤, < 6.0 | 129 | 104 |
| 6.0 ≤, < 7.0 | 75 | 72 |
| 7.0 ≤, < 8.0 | 37 | 52 |
| 8.0 ≤, < 9.0 | 29 | 42 |
| 9.0 ≤, < 10.0 | 1 | 29 |
| 10.0 ≤, < 11.0 | 28 | 11 |
| 11.0 ≤, < 12.0 | 1 | 11 |
| 12.0 ≤, < 13.0 | 5 | 7 |
| 13.0 ≤, < 14.0 | 0 | 7 |
| 14.0 ≤, < 15.0 | 1 | 2 |
| 15.0 ≤ | 1 | 8 |

GC, gastric cancer

Table S2. HRs of ‘large’ and ‘small’ type III GCs compared to type IV GC at each cutoff value and the proportion of the corresponding patients

| Cut off (cm) |  | ‘Large’ type III | | |  | ‘Small’ type III | | |
| --- | --- | --- | --- | --- | --- | --- | --- | --- |
|  |  | HR | 95% CI | Corresponding patients (%) |  | HR | 95% CI | Corresponding patients (%) |
| 4.0 |  | 0.50 | 0.35 – 0.72 | 77.8 |  | 0.22 | 0.13 – 0.37 | 22.2 |
| 5.0 |  | 0.56 | 0.38 – 0.81 | 52.2 |  | 0.31 | 0.21 – 0.47 | 47.8 |
| 6.0 |  | 0.59 | 0.39 – 0.88 | 30.7 |  | 0.37 | 0.25 – 0.54 | 69.3 |
| 7.0 |  | 0.60 | 0.38 – 0.94 | 18.4 |  | 0.40 | 0.28 – 0.58 | 81.6 |
| 8.0 |  | 0.60 | 0.36 – 0.99 | 12.3 |  | 0.41 | 0.29 – 0.59 | 87.7 |
| 10.0 |  | 0.74 | 0.41 – 1.33 | 6.9 |  | 0.42 | 0.29 – 0.59 | 93.1 |
| 12.0 |  | 1.10 | 0.39 – 3.09 | 1.6 |  | 0.43 | 0.30 – 0.61 | 98.4 |

HR, hazard ratio; GC, gastric cancer; 95% CI, 95% confidence interval
